# Genetic risk predictions using deep learning models with summary data

**DOI:** 10.3389/fbinf.2025.1657021

**Published:** 2026-01-08

**Authors:** Angela Wang, Elena Xiao, Jason Cheng, Xiaoxi Shen

**Affiliations:** 1 University School of Milwaukee, Milwaukee, WI, United States; 2 Department of Mathematics, Texas State University, San Marcos, TX, United States; 3 Westwood High School, Austin, TX, United States

**Keywords:** bootstrap, deep neural networks, linkage disequilibrium, risk prediction, single nucleotide polymorphisms

## Abstract

**Background:**

As a driving force of the Fourth Industrial Revolution, deep learning methods have achieved significant success across various fields, including genetic and genomic studies. While individual-level genetic data is ideal for deep learning models, privacy concerns and data-sharing restrictions often limit its availability to researchers.

**Methods:**

In this paper, we investigated the potential applications of deep learning models—including deep neural networks, convolutional neural networks, recurrent neural networks, and transformers—when only genetic summary data, such as linkage disequilibrium matrices, is available. The bootstrap method was used to approximate the test error. Simulation studies and real data analyses were conducted to compare the performance of deep learning methods in genetic risk prediction using individual-level genetic data versus genetic summary data.

**Results:**

The test mean squared errors (MSEs) of most applied deep learning models are comparable when using individual-level data versus summary data.

**Conclusion:**

Our results suggest that suitable deep learning methods could also serve as an alternative approach to predict disease related traits when only linkage disequilibrium matrices are available as input.

## 1 Introduction

With the high throughput of genetic sequencing technology and the success of genome-wide association studies (GWAS), numerous disease-related single nucleotide polymorphisms (SNPs) have been identified in various studies (Consortium, 2007; Scott et al., 2007; Sladek et al., 2007). Additionally, in 2015, President Obama launched the Precision Medicine Initiative, which aims to revolutionize medical treatment for complex diseases (Collins and Varmus, 2015). One of the most critical aspects of precision medicine is customizing treatments based on the unique genetic information carried by each individual. Therefore, accurate risk prediction of disease onset and progression based on an individual’s genetic data can enable targeted preventive treatments (Jostins and Barrett, 2011).

It is widely believed that multiple SNPs contribute to a trait, with the genetic effect arising from the cumulative impact of these genetic variants (Yang et al., 2010). As a result, linear mixed-effects models are conventional tools for genetic risk prediction, where the fixed effects include the clinical and demographic variables (e.g., age and gender) and the effect size of each SNP in a genetic region is treated as a random variable. The total genetic effect is calculated by aggregating the SNPs and their random effects. The Best Linear Unbiased Prediction (BLUP) is a commonly used method for predicting disease-related traits (Campos et al., 2013; Speed and Balding, 2014). However, one limitation of linear mixed-effects models is the assumption that the relationship between SNPs and the trait is strictly linear. In reality, many diseases, such as Alzheimer’s disease (AD), have substantial genetic components and complex genetic etiologies (Karch et al., 2014; Sims et al., 2020). Therefore, it is more reasonable to assume that the relationships between SNPs and disease-related traits are nonlinear. This includes nonlinear relationships between genetic regions such as epistasis as well as those within a genetic region including compound heterozygosity (Nazarian et al., 2023) and allele dosage for AD (Hostage et al., 2013). Although such nonlinearity can be incorporated into a linear mixed-effects model through kernel methods (Hofmann et al., 2008), the performance of BLUP depends on the choice of kernels. In general, it is not clear which kernel is optimal for prediction. In this manuscript, the hippocampal volume is used as the phenotype in the real data analysis. The motivation for this choice stems from previous findings. First, the hippocampus plays a crucial role in memory and is particularly vulnerable to damage at the early stages of Alzheimer’s disease (AD) (Mu and Gage, 2011). Changes in hippocampal volume over time can have a significant impact on AD progression (Schuff et al., 2009). Accurately predicting hippocampal atrophy could therefore facilitate early intervention in disease development. Moreover, the genetic influence on hippocampal volume is relatively high. For instance, in a study of a large sample of elderly twin men, (Sullivan et al., 2001) showed that approximately 40% of the variance in hippocampal volume is attributable to genetic factors. In addition, an exploratory GWAS of hippocampal volume using data from the Sydney Memory and Ageing Study suggested that the heritability of hippocampal volume is 62%–65% (Mather et al., 2015). Second, the relationship between SNPs and hippocampal volume is potentially complex. For instance, studies have identified interactions between rs1345203 and rs1213205 that explain 1.9% of the variance in temporal lobe volume (Hibar et al., 2015). The ability of deep learning methods to capture nonlinear relationships may help improve predictive performance in this context. As an example illustrating the predictive performance of deep learning models based on genetic data, (Liu et al., 2022) used a deep neural network–based model to predict AV45 and FDG, two tracers in positron emission tomography (PET) imaging used to model biological processes in the brain, based on genetic data. Compared with other mixed-effects model–based methods, the deep neural network–based models achieved better prediction accuracy.

Since 2010, advancements in deep learning technologies have become the driving force behind the fourth Industrial Revolution (Bai et al., 2020). With numerous successful applications, such as in computer vision and natural language processing, deep neural networks (DNNs) have emerged as one of the most popular research tools across various scientific fields. A major advantage of DNNs is their ability to capture complex relationships between variables, thanks to the universal approximation property (UAP) (Cybenko, 1989; Hornik et al., 1989). This makes them suitable candidates for approximating the complex relationships between genetic variants and disease-related traits. On the other hand, kernel methods have also been widely used in genetic studies to capture the nonlinear relationships (Hofmann et al., 2008). However, the performances of kernel methods highly depend on whether the kernel function has been chosen wisely as well as the specification of hyperparameters in the kernel function (e.g., the degree in a polynomial kernel). Compared to kernel methods, one only needs to specify the number of layers and the number of hidden units in a layer to construct a DNN, which from our point of view, is easier than selecting the correct kernel function. A lot of research has been conducted to uncover complex genotype-phenotype relationships using DNNs. For example, DNNs have been used to model Alzheimer’s disease (AD) polygenic risk, outperforming traditional methods (Zhou et al., 2023). Additionally, Shen and Wang, 2024 employed deep ReLU neural networks to detect significant SNPs associated with phenotypes. Their simulation studies demonstrated that tests based on deep ReLU neural networks are more powerful at detecting nonlinear relationships compared to F-tests in linear models. We refer interested readers to Shen et al. (2022b) for a review of applications of deep learning models in genetic and genomic studies.

Although individual-level genetic data can improve the precision of predictions, such data is often difficult to obtain due to privacy concerns and data-sharing restrictions. Recently, many researchers have focused on conducting analyses using GWAS summary data, including gene- and pathway-based association tests (Guo and Wu, 2019; Kwak and Pan, 2016; Svishcheva et al., 2019), genetic heritability estimations (Li et al., 2023; Speed et al., 2020; Speed and Balding, 2019), and the detection of causal associations (Xue and Pan, 2020; Zhu et al., 2018). The goal of this paper is to explore whether deep learning methods, such as convolutional neural networks (CNNs) (LeCun, 1989) and long short-term memory networks (LSTMs) (Hochreiter and Schmidhuber, 1997), can achieve predictive performance on genetic data comparable to that obtained when applying the same model structures to individual-level data.

The rest of the paper is organized as follows: In the Methods section, we briefly review basic deep learning models, including deep neural networks, convolutional neural networks, and recurrent neural networks, such as LSTMs. We then propose a framework for applying deep learning models to GWAS summary data (e.g., the linkage disequilibrium (LD) matrix) and outline the approach for calculating the test error. The Results section presents simulation studies and an application predicting Alzheimer’s disease-related traits using real data from the Alzheimer’s Disease Neuroimaging Initiative (ADNI). Finally, we conclude with a discussion of the proposed method and its potential future improvements.

## 2 Methods

### 2.1 Simulation data

To evaluate the proposed methodologies, extensive simulation studies were conducted. The simulations involved applying DNN, CNN, LSTM and Transformers to both individual-level genetic data and summary data (i.e., LD matrix). The training errors and testing errors obtained using the summary data were compared with those from using the individual-level data. The data used for simulations was generated using R and the implementation of the deep learning methods was conducted using the Keras package in python.

#### 2.1.1 Individual-level data

To mimic the real structure of genetic sequencing data, the data used for simulation were generated based on the real sequencing data from Chromosome 17: 7344328-8344327 in the 1,000 Genomes Project (The 1000 Genomes Project Consortium, 2010). The minor allele frequencies (MAF) of the SNPs in this region range from 0.046% to 49.954%. Since deep learning models have better performance when the signal is strong (James et al., 2013), including rare SNPs could deteriorate the performance. Therefore, we removed SNPs with MAF <0.001 were removed. Each remaining SNP was scaled to have a sample mean of 0 and a sample standard deviation of $1/\sqrt{p}$ , where *p* = 8,299 is the number of common SNPs in this region. To simulate the response variable, 30% of the common SNPs were randomly selected as causal variants, and the response variable was generated using the following equation:

$$Y_{i}=\sum_{k=1}^{K}w_{k}g_{i,k}+\varepsilon_{i},i=1,\ldots ,n,$$

where *K* is the number of causal variants; $g_{i,k}$ is the value of the *k*th causal SNP for the *i*th individual and $w_{1},\ldots ,w_{K}∼i.i.d.\;N\left(0,0.6^{2}\right)$ and $\varepsilon_{1},\ldots ,\varepsilon_{n}∼i.i.d.\;N\left(0,1\right)$ . The dataset consists of *n* = 1,092 individuals. When training deep learning models, 80% of the samples were randomly selected as training data and the remaining samples were used as test data.

#### 2.1.2 Construction of LD matrices

In terms of the summary data, the LD matrices were generated as follows. Let *G* denote the scaled SNP data as mentioned above, which contains 1,092 individuals and 8,299 SNPs. To generate the LD matrix for training the deep learning models, 80% of the samples were randomly selected, and the SNPs of the remaining individuals were used to generate the LD matrix for testing purposes. Let $G_{tr}$ be the design matrix of the scaled SNPs in the training data and $G_{te}$ be the design matrix of the scaled SNPs in the test data. In other words, $G_{tr}$ was extracted from *G* by taking the rows corresponding to the training samples and $G_{te}$ contains the remaining rows in *G*. The LD matrices for training and testing were generated by calculating $\frac{1}{n_{tr}}G_{tr}^{T}G_{tr}$ and $\frac{1}{n_{te}}G_{te}^{T}G_{te}$ , respectively. Here $n_{tr}=873$ represents the number of training data and $n_{te}=219$ represents the number of test data.

### 2.2 Real data

Alzheimer’s disease (AD) is one of the most common neurodegenerative diseases, significantly influenced by genetic factors (Karch et al., 2014; Sims et al., 2020). Effective predictions on the development of AD based on genetic components could lead to early intervention as well as targeted treatment of the disease. In this section, we applied the proposed methods to perform genetic risk prediction using deep learning models on a real dataset from the Alzheimer’s Disease Neuroimaging Initiative (ADNI (https://adni.loni.usc.edu/)). The ADNI study is a multisite, longitudinal observational study aimed at improving the scientific understanding of Alzheimer’s disease (AD). Data from phases 1 and 2 of the ADNI study were used in the analysis. A total of 3,108 participants were included across these two phases, with about 89% of the participants identifying as white and an average baseline age of 72.76 years. More demographic information is summarized in Table 1.

**TABLE 1:** Demographics of participants in ADNI1 and ADNI2 studies.

Demographic variable	Mean (standard deiviation)/Relative frequency
Age	72.76 (7.7)
Years of education	15.4 (3.99)
Gender	
Male	53.15%
Female	43.98%
Self identified race	
American indian or alaskan native	0.06%
Asian	1.54%
Native Hawaiian or other pacific islander	0.06%
Black or african american	4.63%
White	89.25%
More than one race	0.68%
Unknown	0.35%
Self identified ethnicity	
Hispanic or latino	3.09%
Not hispanic or latino	92.82%
Unknown	0.61%
Participant’s primary language	
English	93.56%
Spanish	1.51%
Other	1.58%

A total of 808 samples at the screening and baseline of the ADNI1 and ADNI2 studies have the whole genome sequencing data, and we used SNPs from the *APOE* gene located on Chromosome 19: 45409005-45412652. Variants with a call rate <99% or a Hardy-Weinberg equilibrium p-value < 1e-6 were removed. The final SNP dataset included 780 individuals and 168 SNPs. Regarding the choice of the response variable, the hippocampus region was selected because it plays a vital role in memory (Mu and Gage, 2011), and shrinkage in the hippocampal volume is an early symptom of AD (Schuff et al., 2009). Thus, the volume of the hippocampus region was chosen as a potential response variable. In this dataset, the hippocampal volume has mean 6778.89 with a standard deviation of 1179.07. We first took the logarithm of the hippocampal volume such that it has an approximately normal shape. To remove some confounding effects, we regressed the logarithm of hippocampal volume onto important predictors, including age (mean: 73.46, sd: 7.01), gender (Male: 35.98%, Female: 63.94%), and number of years in education (mean: 16.08, sd: 2.78). The residuals obtained from this regression were used as the response variable to train the deep learning models.

Similar to the simulation studies, 80% of the data were randomly selected as the training data, and the remining data was served as the test data to evaluate model performances. The LD matrices for training and testing were obtained using the matrix inner product of the SNP matrix corresponding to the *APOE* gene in the training set and test set, respectively. The formula of generating the LD matrices is the same as those described in the simulation data. The training data consists of 624 individuals while the test data has 156 individuals.

### 2.3 Deep neural networks (DNNs)

The development of deep neural networks can be traced back to 1950s when a mathematical model, known as the perceptron (Rosenblatt, 1958), was proposed to model the functionality of neurons in a human brain. Around the 1990s, multiple perceptrons were stacked to create artificial neural networks. An artificial neural network consists of three layers: the input layer contains all the features from the data used to make predictions; the hidden layer contains several units to further extract useful information from the inputs, and the output layer produces the output of the artificial neural network to make predictions. Deep neural networks are obtained by including multiple hidden layers. One of the major advantages discovered for DNN is their ability to capture these complex relationships due to their famous universal approximation property (UAP) (Cybenko, 1989; Hornik et al., 1989; Yarotsky, 2017; Yarotsky and Zhevnerchuk, 2020). This suggests that DNNs may be suitable candidates for approximating the complex structures present in such research problems. Because of the UAP, deep neural networks are popular tools in predictive analyses nowadays.

**FIGURE 1:**
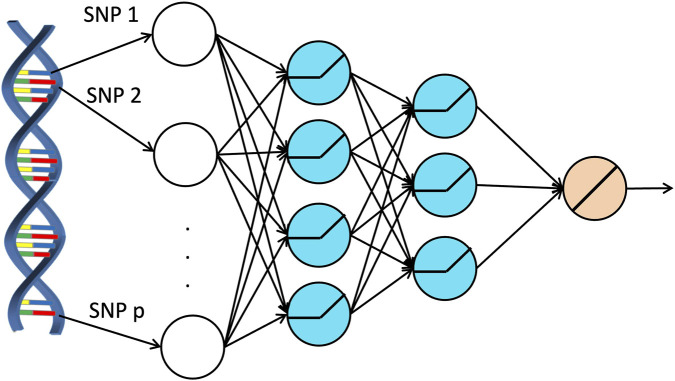
Architecture of a deep neural network applied on genetic data.

Since SNP data and LD matrices often contain nonlinear, high-dimensional relationships that are difficult to capture with classical models. Deep neural networks are well suited because stacked nonlinear layers can learn complex interaction patterns without requiring specifying the functional form of underlying relationship explicitly. The flexibility of DNNs makes them effective when the relevant predictive structure is distributed across many genetic regions. In the application of DNNs to genetic risk prediction, each input unit represents a SNP in a genetic region (e.g., a gene). Throughout the remainder of the paper, additive coding was used for the genotypes (i.e., 0 for genotype AA, 1 for genotype Aa, and 2 for genotype aa) in the raw genetic data. In additive coding, the alleles assigned to ‘A’ and ‘a’ are not arbitrary. ‘A’ always represents the allele with a higher frequency in the population (the major allele), and ‘a’ represents the allele with a lower frequency in the population (the minor allele). In other words, the numerical value used for encoding a genotype in additive coding corresponds to the number of minor alleles in the genotype. Additive coding has been commonly used in the statistical genetics literature (Wu et al., 2011; Li et al., 2014; Shen et al., 2022c). As mentioned in the Simulated Data and Real Data sections, each column of the genotype matrix, which represents a SNP, will be standardized so that the column has mean 0 and standard deviation $1/\sqrt{p}$ with *p* being the number of SNPs. In other words, the actual SNP coding used in the analysis are $\frac{0-m}{s\sqrt{p}},\frac{1-m}{s\sqrt{p}},$ and $\frac{2-m}{s\sqrt{p}}$ with *m* being the column mean and *s* being the column standard deviation. The genetic information then passes through hidden layers to extract important features from the data. The output layer, which contains a single unit with a linear activation function, produces the predicted value of the trait. Figure 1 provides a graphical illustration of the structure of a deep neural network.

When applying DNNs to the simulated and the real individual level SNP dataset, each SNP was used as an input. In other words, the input dimension is 8,299 for the simulated data and is 168 for the real data. We used a similar structure as described in Zhou et al. (2023), with the network comprising four hidden layers containing 231, 77, 22, and 5 hidden units, respectively. Additionally, one dropout layer with dropout rate 0.2 was added after the first hidden layer and another dropout layer with dropout rate 0.5 was added after the third hidden layer. When the DNNs were trained using the adaptive moment estimation (ADAM) (Kingma and Ba, 2017), we set the number of epochs to 100, the batch size to 256, and the learning rate to 0.001 with a decay rate of 0.96. These hyperparameters were selected based on validation errors from a predefined set of candidate values.

### 2.4 Convolutional neural networks (CNNs)

CNNs are a class of neural networks that use filters to process multidimensional data, such as images, by extracting relevant features. The core building blocks of CNN include convolutional layers and pooling layers, which work together to refine feature representations (Li et al., 2022).

**FIGURE 2:**
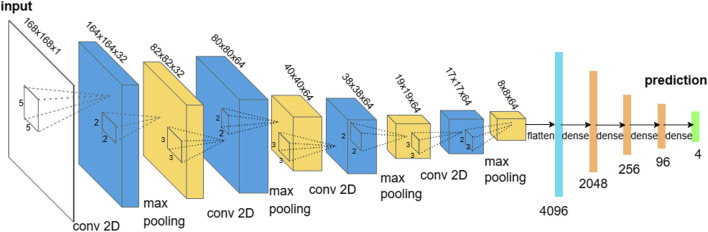
Structure of a convolutional neural network. The input is a 2-dimensional array (in our applications, it could be a SNP matrix or a LD matrix). The blue cuboid and the yellow cuboid represent convolutional layers and max-pooling layers respectively. After the last max pooling layer, the features were fed to a fully connected deep neural network to make predictions.

Each convolutional layer consists of several filters, with each filter acting as a sliding window that applies a nonlinear activation function (such as ReLU) to the linear combination of filter entries and outputs from the previous layer, producing feature maps. Pooling layers reduce the size of the representation, accelerating computations and enhancing the robustness of detected features. A commonly used pooling technique is max pooling, which extracts the maximum value within a sliding window. After passing through multiple convolutional and pooling layers, the extracted features are flattened into a vector and fed into a fully connected neural network to make final predictions. Figure 2 illustrates the basic structure of a convolutional neural network.

Additionally, the weights in the filters of convolutional layers and in the fully connected neural network are trained using backpropagation, with the learning rate controlling the rate of parameter updates. However, CNNs may encounter issues such as vanishing or exploding gradients during training. To improve generalization and reduce overfitting to the training data, dropout layers can be employed. These layers randomly deactivate nodes during training, preventing the model from relying too heavily on specific neurons. This encourages the development of more general and robust models. While CNNs excel at capturing local patterns, they may struggle with broader patterns, which require additional and larger-size filters. However, increasing filter sizes and quantity could significantly raises computational cost and training time.

Due to the fact that CNNs can capture local patterns, it makes CNNs a natural fit when the SNPs exhibit spatial structure along the genome. In particular, adjacent SNPs tend to be correlated due to LD, so a CNN’s convolutional filters can detect local patterns or LD blocks similarly to how they detect edges or textures in images. Moreover, weight sharing dramatically reduces the number of parameters, helping the model generalize even when training data are limited. This makes CNNs especially useful for modeling local genetic architecture and short-range dependencies. When applying CNNs to simulated and real individual level SNP data, the inputs are the values of each SNP and 1-dimensional filters were used (i.e., the input dimension is 8,299 for the simulated data and is 168 for the real data). The rationale for using 1-dimensional filters for individual-level data is that individuals are typically considered independent observations, a common assumption in machine learning theory. As a result, local information is only present within the observations of the SNPs. Based on validation errors from a predefined set of candidate hyperparameter values using 1-dimensional filters, two CNN structures outperformed the others. The first structure consists of one convolutional layer with 50 filters, each of size 500, and one hidden neural network layer with 50 hidden units. The second structure includes one convolutional layer with 50 filters, each of size 500, and five hidden neural network layers, each with 50 hidden units. In all cases, each hidden layer used a ReLU activation function. Training was conducted over 200 epochs with a batch size of 32, an initial learning rate of 0.1, and a decay rate of 0.98.

### 2.5 Recurrent neural networks (RNNs)

**FIGURE 3:**
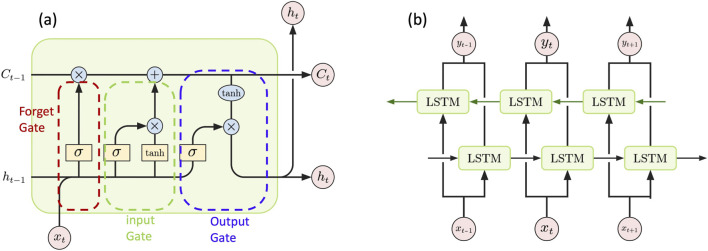
**(a)** Structure of an LSTM unit where $x_{t}$ is the current input of a LSTM unit; $h_{t-1}$ and $h_{t}$ are the outputs from the previous LSTM unit and the current LSTM unit respectively; $C_{t-1}$ and $C_{t}$ represent the memories from the previous LSTM units and the updated memory after the current LSTM unit, respectively. Additionally, σ and tanh are the sigmoid and hyperbolic tangent activation function. The unit consists of three main gates: the forget gate, the input gate, and the output gate. The forget gate uses a dense layer with inputs $x_{t}$ and $h_{t-1}$ to determine what proportion of previous information should be discarded. The input gate consists of two parts: the first is a dense layer with a sigmoid activation function, and the second is another dense layer with a hyperbolic tangent activation function. Both dense layers take $x_{t}$ and $h_{t-1}$ as input. The first part determines how much new information $x_{t}$ contributes to the cell and the second part provides a candidate cell state. The output gate, which also consists of a dense layer, controls what information from the cell state is used to compute the final output. **(b)** A BiLSTM contains both forward and backward loops to improve the model for capturing long-rage dependencies. In the figure each box of LSTM represents a single LSTM unit with the structure shown in **(a)** Here $x_{t-1},x_{t},x_{t+1}$ form a segment of input sequence and $y_{t-1},y_{t},y_{t+1}$ are the corresponding output sequence.

RNNs are a class of neural networks extended to include feedback connections. This allows them to capture temporal patterns in sequential data. The long-short term memory (LSTM) is a special type of RNN aimed at learning long-range dependencies by mitigating the vanishing gradient problem that traditional RNNs struggle with (Bengio et al., 1994). An LSTM unit uses input, forget, and output gates to control the flow of information into and out of its memory cell. This structure allows the network to retain relevant information across longer time spans. Figure 3a provides an illustration of the structure of an LSTM unit.

Due to linkage disequilibrium, SNPs are often correlated, making LSTM an ideal model for capturing sequential dependencies in genetic data. In addition, the physical positions of SNPs reflect the underlying linkage disequilibrium (LD) structure and local genomic context, as nearby SNPs often exhibit correlated variation due to shared inheritance. By leveraging its memory cells and gating mechanisms, LSTM can be trained on numerical SNP sequences, which are the ordered sequences of SNP genotypes encoded as 0, 1 and 2, representing the number of minor alleles at each locus, to learn complex temporal and spatial dependencies in genomic structures. This capability makes it particularly effective for identifying nonlinear relationships between correlated genetic features and response variables. In our project, we numerically encoded SNPs as inputs for models composed of multiple LSTM units and trained these models to predict disease-related traits.

To further improve the models’ ability to capture long-range dependencies, we also implemented a bidirectional approach. Unlike unidirectional LSTMs, which only process information in the forward direction, bidirectional LSTM (BiLSTM) networks traverse the input data both forwards and backwards. This allows them to produce outputs based on later context, while LSTM relies only on previous context. As a result, BiLSTM networks generally outperform LSTM (Graves and Schmidhuber, 2005; Siami-Namini et al., 2019). The structure of a BiLSTM is described in Figure 3b.

Similar to the DNN and CNN models, the inputs for the LSTM and BiLSTM models are the SNP values in the data. Hence, the input dimensions for the simulated data and the real data are 8,299 and 168, respectively. When applying LSTM to individual-level SNP data, we used a five-layer LSTM, with each layer containing 10 LSTM units, followed by a dense layer. Training was conducted over 10 epochs with a batch size of 256. For the BiLSTM, we used a two-layer architecture, with each layer containing 10 BiLSTM units, followed by a dense layer. The model was trained for 5 epochs with a batch size of 256. For both LSTM and BiLSTM models, the ADAM optimizer was used with an initial learning rate of 0.001 and a decay rate of 0.96. These hyperparameters were determined based on validation errors from a predefined set of candidate values.

### 2.6 Transformers

**FIGURE 4:**
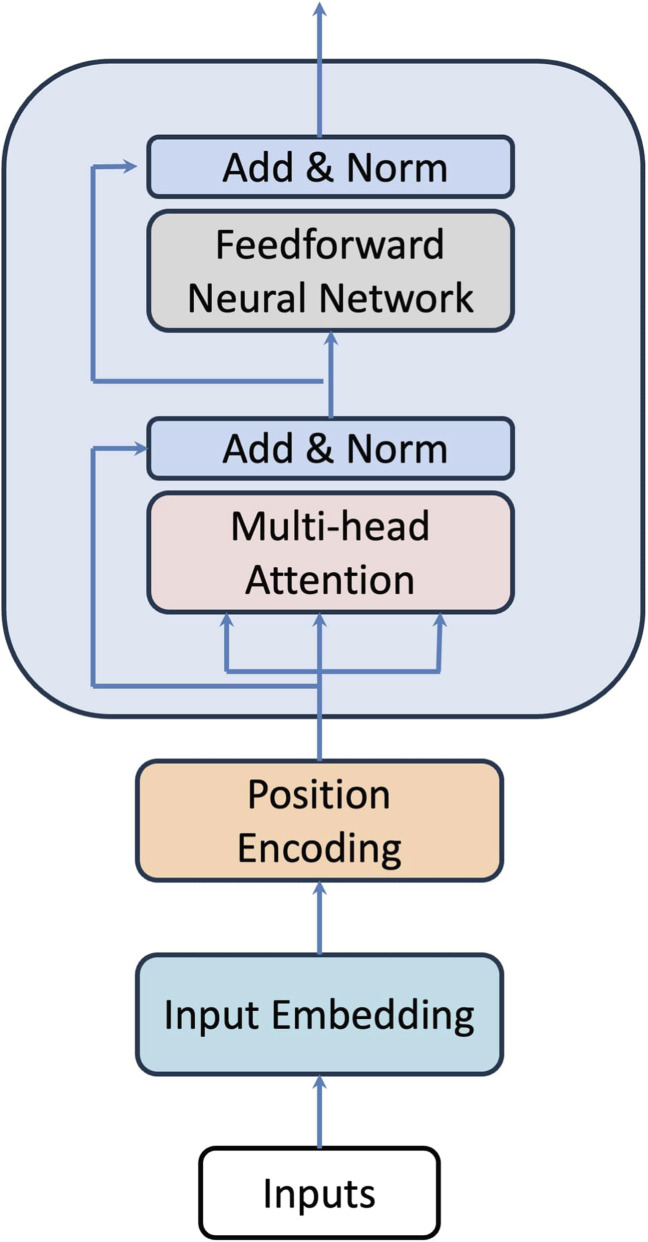
Structure of a transformer encoder. There are four main components in the structure. Input embedding converts categorical inputs (e.g., each word or symbol in the vocabulary) into numerical vectors. Positional encoding allows the transformer to keep track of the order of words in a sequence. Multi-head attention computes the relationships between each word and all other words in the sentence, including itself and the residual connections provide shortcut paths that help stabilize and speed up the training of deep networks.

The transformer architecture (Vaswani et al., 2017) brings deep learning into a modern era. Although the original work focused on English–German machine translation, transformers have since been widely applied to a broad range of tasks, including applications in genetics and genomics (Graça et al., 2024; Li et al., 2025). A transformer model consists of an encoder and a decoder. In our application, only the encoder component was used. The structure of the transformer encoder is illustrated in Figure 4. There are four main components in a transformer encoder:

1. Input embedding converts categorical inputs (e.g., each word or symbol in the vocabulary) into numerical vectors.
2. Positional encoding allows the transformer to keep track of the order of words in a sequence.
3. Multi-head attention computes the relationships between each word and all the words in the sentence, including itself.
4. Residual connections provide shortcut paths that stabilize and speed up the training of deep networks.

Since transformers use self-attention to model relationships between all pairs of positions in a sequence simultaneously, it makes them highly effective for genetic data where both local and long-range LD patterns matter. Instead of processing the sequence step by step, transformers directly learn how each SNP is related to others. In addition, interactions across the genetic region or LD matrix are modeled more flexibly, making transformers particularly powerful for discovering complex relationships across the genetic regions. On the other hand, because of the high dimensionality of SNP data (e.g., the simulated dataset contains 8,299 SNPs), feeding all SNPs into a transformer simultaneously would result in excessive memory usage and computational cost. To address this issue, we applied a sliding window of size 1,000 with a stride of 500, generating 15 overlapping SNP sequences of length 1,000. Each of these SNP sequences was used as input to the transformer, and the outputs from the multi-head attention units were averaged to form the input to the final feedforward neural network. The number of multi-head attention units was set to 6, and the number of hidden units in the final feedforward neural network was 64. The ADAM optimizer was used with a batch size of 8 and 10 training epochs.

### 2.7 Applications to summary data

Although deep learning models are expected to perform better on individual-level genetic data, such data are not always accessible due to privacy concerns and data-sharing restrictions. In contrast, genetic summary data are more readily available, making it worthwhile to investigate the performance of deep learning models on this type of data. Additionally, it is important to assess whether prediction accuracy based on genetic summary data is comparable to that achieved with individual-level data. In this paper, we utilized the LD matrix as our summary data. Although other GWAS summary statistics can also help address privacy concerns, using LD matrices as summary data offers additional benefits. GWAS summary statistics, such as polygenic risk scores (PRS), are calculated based on the marginal linear effects of SNPs. As a result, information about the interactions and correlations among SNPs is not taken into account, whereas LD matrices preserve these correlations and can improve predictive performance.

**FIGURE 5:**
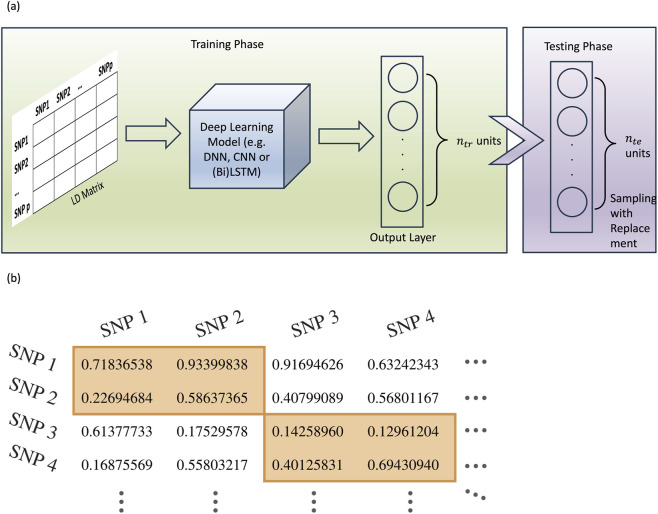
Illustration of application of deep learning models to genetic summary data. **(a)** In the training phase, the LD matrix of a genetic region, derived from the training data, is used as input. Deep learning models such as DNN, CNN, or LSTM are then applied to this input LD matrix. The number of output units of these deep learning methods is set to be equal to the number of training samples. In the testing phase, the LD matrix from the test data will first be fed to the trained deep learning model. $n_{te}$ outputs are then randomly selected with replacement from the $n_{tr}$ outputs. Denote the test error based on this random sample as $TE_{1}$ . This process is then repeated *B* times to obtain a set of bootstrapped test error samples $TE_{1},\ldots ,TE_{B}$ . The approximated test error will be the mean of these *B* test errors. **(b)** In the situation of large LD matrix, to reduce computational burden, the LD matrix will be broken into smaller submatrix along the diagonal (as shown in the orange box). These smaller LD matrices will then be used as inputs to train deep learning models.

A significant challenge in applying deep learning models to genetic summary data is evaluating the prediction error on both the training and, more importantly, the test data. Our proposed approach is illustrated in Figure 5a. During the training phase, the LD matrix of a genetic region, derived from the training data, is used as input. Deep learning models such as DNN, CNN, or LSTM are then applied to this input LD matrix. Unlike the traditional approach, where the number of units in the output layer matches the dimension of the response variable, this framework sets the number of output units equal to the number of training samples. Let $Y_{1},\ldots ,Y_{n_{tr}}$ be the quantitative response variables in the training set and let $O_{1},\ldots ,O_{n_{tr}}$ be the outputs from a deep learning model, the parameters in a deep learning model were trained via ADAM to minimize the loss function

$$L\left(O,Y\right)=\frac{1}{n_{tr}}\sum_{i=1}^{n_{tr}}{\left(O_{i}-Y_{i}\right)}^{2}.$$

To evaluate the test error, we adopted the resampling approach from the well-known bootstrap method (Efron and Tibshirani, 1994). First, the LD matrix from the test data will be fed to the trained deep learning model. Once the $n_{tr}$ outputs are obtained, we randomly select $n_{te}$ outputs with replacement from these outputs. Denote the test error based on this random sample as $TE_{1}$ . This process is then repeated *B* times to obtain a set of bootstrapped test error samples $TE_{1},\ldots ,TE_{B}$ . The approximated test error will be the mean of these *B* test errors:

$$\hat{TE}=\frac{1}{B}\sum_{j=1}^{B}TE_{j}.$$

The following network structures were used when applying the deep learning models to LD matrices:

- *DNN*: The elements in the upper triangle of the LD matrix were used as inputs for the DNN. The network structure was the same as that used for the individual-level SNP data. The ADAM optimizer was applied, and we set the number of epochs to 100, the batch size to 256, and the learning rate to 0.001 with a decay rate of 0.96.
- *CNN*: Since LD matrices are inherently two-dimensional, and the inputs used in CNNs are typically images, we treated the LD matrices as “images” when applying CNNs. Unlike the CNN models for individual-level SNP data, two-dimensional filters were applied when the inputs were LD matrices. We then used the same CNN architecture as that described for the individual-level SNP data. Due to the structure of CNNs, the size of the two-dimensional filters was scaled down by a factor of 10 compared to their one-dimensional counterparts. In other words, two-dimensional filters of size 50 × 50 were used instead. This scaling was necessary because most CNN models with two-dimensional filters could not complete training within a reasonable time frame. Training was conducted over 200 epochs with a batch size of 32, an initial learning rate of 0.1, and a decay rate of 0.98 per epoch.

For the real data, since the LD matrix based on the *APOE* gene is significantly smaller than the one used in the simulation studies, we modified the convolutional layers in the two CNN structures. The first structure consists of one convolutional layer with 32 filters, each of size 5, followed by a pooling layer with a pooling size of 2. The second structure includes four convolutional layers: the first with 32 filters of size 5, followed by three layers, each with 64 filters of size 3. Each convolutional layer is followed by a max pooling layer with a pooling size of 2. For all configurations, each hidden layer employed a ReLU activation function. The same CNN architectures were then applied to the summary-level data using two-dimensional (2D) convolutional filters with sizes 5 × 5 and 3 × 3, along with max pooling layers having filters of size 2 × 2.

- *LSTM/BiLSTM*: When the LSTM or BiLSTM models were applied to the LD matrices, the entire LD matrix was used as input. In other words, the input dimensions were 8,299 × 8,299 for the simulated data and 168 × 168 for the real data. The same LSTM and BiLSTM architectures used for the individual-level SNP data were also applied to the LD matrices. In addition, the ADAM optimizer was used with 10 epochs and a batch size of 256 for the LSTM, and with 5 epochs and a batch size of 256 for the BiLSTM. For both models, the initial learning rate was set to 0.001, with a decay rate of 0.96 per epoch during training.
- *Transformer*: Due to the large size of the LD matrix in the simulated data, the transformer input consisted of diagonal blocks extracted from the original LD matrix, as described in the following paragraph. Without this operation, training a transformer would be infeasible because of the tremendous memory requirements. For the real data, we directly used the 168 × 168 LD matrix as the input. The input data were first flattened and embedded into a vector of dimension 128. Positional encoding was then applied, followed by two transformer blocks, each containing six multi-head attention units, a dropout layer with a dropout rate of 0.1, and layer normalization. Finally, two dense hidden layers, each with 128 hidden units, were applied, followed by another dropout layer (dropout rate 0.1) and layer normalization.

Training a deep learning model, especially a CNN or a transformer with an LD matrix as input, can involve a large number of parameters. In addition, when the dimension of the LD matrix is large, storing such a large LD matrix requires substantial memory. Combining with the number of parameters to train in a deep learning model, it could lead to prohibitively long computation time. To reduce the computational burden in cases with large LD matrices, the matrix is divided into smaller block matrices along the diagonal, and these smaller LD matrices are used as inputs instead. This idea is originated from the fact that LD is largely local due to the haplotype block structure of SNPs (Gabriel et al., 2002) and is illustrated in Figure 5b. Although dividing the LD matrix into smaller blocks may lead to the loss of some long-range SNP relationships, doing so helps mitigate substantial computational challenges. Moreover, when multiple small LD blocks are fed into a deep learning model, such as a convolutional neural network (CNN), the model can still capture correlations between neighbouring blocks, which may partially recover the missing LD information. In our application, a block size of 193 was used so that 8,299 SNPs resulted in exactly 43 smaller blocks, and these blocks of LD matrices were used as inputs when training the CNN and the transformer.

When applying deep learning models with LD matrices as inputs, there is an inherent trade-off between retaining genomic information and managing computational cost. For the DNN and LSTM models, we used the entire LD matrix as input, as the computational costs of DNN and LSTM are less demanding. In DNN applications, we used the upper triangular portion of the LD matrix, which does not result in information loss because the LD matrix is symmetric. In contrast, for the CNN and transformer models, which are more computationally intensive, it should be noted that we used the same block LD matrices along the diagonal as inputs to reduce computational burden.

## 3 Result

Table 2 summarizes the training and test errors of the deep learning models applied to individual-level SNP data as well as to LD matrices. The results were obtained from 500 independent repetitions. Each cell contains the sample mean of the training/test errors based on the 500 runs, with the sample standard deviation provided in parentheses. As shown in Table 2, when individual-level SNP data is available, the training errors from the DNN and CNN (structure 1) are relatively small, but their test errors are larger. Notably, the test errors of CNNs are significantly higher than those of DNNs, suggesting that CNNs may be less effective for SNP data. In contrast, both LSTM and BiLSTM models exhibit smaller test errors compared to DNNs, indicating that LSTM-based models may be more suitable for SNP data. In addition, the transformer models achieved a performance comparable to that of the LSTM and BiLSTM on the individual-level SNP data, but the test error was higher when only the LD matrix was available. A key observation from Table 2 is that when only genetic summary data (LD matrices) is available, deep learning models can still achieve performance comparable to that obtained with individual-level SNP data.

**TABLE 2:** Comparisons between training/test errors of deep learning models on individual-level SNP data and on genetic summary data based on simulated data.

Method		Individual-level SNP data		Genetic summary data	
		Training error	Test error	Training error	Test error
DNN		0.564 (3.720e-01)	1.233 (8.589e-02)	0.119 (9.714e-02)	1.822 (1.758e-01)
CNN					
	Structure 1	0.356 (3.237e-01)	1.466 (2.198e-01)	0.694 (2.190e-02)	2.303 (6.369e-04)
	Structure 2	1.338 (5.434e-01)	1.367 (6.085e-01)	0.720 (1.149e-02)	2.303 (6.375e-04)
LSTM		1.119 (1.034e-04)	1.134 (4.835e-03)	1.109 (1.945e-03)	1.153 (4.573e-04)
BiLSTM		1.119 (1.471e-04)	1.136 (5.407e-03)	1.101 (2.720e-04)	1.153 (2.171e-04)
Transformer		1.120 (2.358e-02)	1.118 (9.446e-02)	0.182 (1.320e-02)	1.894 (2.225e-02)

Table 3 summarizes the results obtained from the real data analysis. Similar to the simulation studies, 500 independent runs were performed on different random initialization and the training/test errors have a very similar pattern as in Table 2. The performances of all deep learning models applied on LD matrices are similar to those obtained from using individual-level SNP data. On the other hand, both BiLSTM and CNN perform better compared to that of DNN’s and CNN performs the best. We hypothesize that SNPs within the *APOE* gene are more spatially correlated compared to the ones generated in the simulation studies. It is a little bit surprise to see that transformers did not perform very well in this case as the test error is significantly larger than other statistical models, which could potentially be due to the small sample size in the ADNI data.

**TABLE 3:** Comparisons between training/test errors of deep learning models on individual-level SNP data and on genetic summary data based on ADNI data. The unit for the response variable (logarithm of hippocampal volume) is the natural logarithm of cubic millimeters.

Method		Individual-level SNP data		Genetic summary data	
		Training error	Test error	Training error	Test error
DNN		2.188e-02 (1.923e-03)	2.118e-02 (1.118e-03)	3.494e-03 (1.272e-03)	1.694e-02 (2.237e-04)
CNN					
	Structure 1	1.389e-02 (8.903e-4)	1.397e-02 (9.313e-4)	1.430e-02 (3.564e-3)	1.531-e02 (2.412e-04)
	Structure 2	1.389e-02 (4.448e-4)	1.391e-02 (3.936e-4)	1.356e-02 (5.1131e-4)	1.389-e02 (1.863e-04)
LSTM		2.345e-02 (6.690e-05)	2.063e-02 (2.266e-04)	2.265e-02 (3.367e-04)	2.265e-02 (4.052e-04)
BiLSTM		2.364e-02 (4.794e-04)	2.059e-02 (4.054e-04)	1.407e-02 (1.537e-04)	1.392e-02 (7.873e-06)
Transformer		1.532e-02 (4.477e-06)	1.394e-02 (4.304e-06)	2.392e-02 (2.663e-03)	5.495e-02 (2.959e-03)

As a comparison, we also applied the best linear unbiased predictor (BLUP) from linear mixed-effects models to predict the traits based on the simulated individual-level SNP data, which serves as a benchmark. Based on 500 independent runs, for the simulated data, the mean and standard deviation of the training error of BLUP were 0.849 and 3.499e-01, respectively, and the mean and standard deviation of the test error were 1.087 and 9.984e-02, respectively. Although BLUP appears to outperform all the deep learning models due to its smaller test error, it should be noted that the underlying relationship between the SNPs and the trait is linear. Therefore, BLUP is expected to be the best predictor in terms of mean squared error.

## 4 Discussions and conclusion

Deep learning methods have achieved significant success in genetic and genomic predictive analyses. However, their application to genetic summary data has not been fully explored. In this paper, we propose an approach for training and evaluating deep learning models using genetic summary data as inputs, with the test error approximated through the bootstrap method. Through simulation studies and real data analyses, we find that deep learning methods based on LD matrices can achieve prediction accuracies comparable to those obtained using individual-level data. This finding broadens the potential applications of deep learning methods to genetic risk prediction based on summary data.

In practice, the performance of deep learning methods heavily depends on the choice of hyperparameters (such as the number of hidden units and hidden layers in a DNN) as well as the learning algorithms. To determine these hyperparameters, we created a pool of deep learning models with varying configurations, selecting those with the best validation prediction accuracy. Developing effective strategies for choosing hyperparameters to ensure optimal predictive performance will be a focus of our future work. Additionally, while CNNs can capture local information, it is noteworthy that their performances vary a lot in the simulation studies and the real data analyses. We conjecture that the performance of CNNs relies heavily on the noises and spatial correlations of SNPs in the genetic data, which could potentially limit their feature extraction capabilities. Further analyses on different simulated and real genetic datasets are needed to verify this conjecture, and this will be another work of our future research. On the other hand, the performances of LSTM or BiLSTM are more consistent and can produce slightly better test error compared to DNNs, which may suggest these models are more suitable for genetic and genomic applications.

Computational costs and limited sample sizes are two major bottlenecks of the proposed method. To enable a deep learning model to flexibly capture complex relationships, larger network architectures and greater sample sizes are preferred. However, due to limited sample sizes and computational resources, we were only able to test deep learning models with relatively small architectures. Even with such small structures, it remains infeasible to use a genome-wide LD matrix as input. In the paper, we proposed to use nonoverlap block matrices along the diagonal to address the issue. To avoid further information loss, a potential solution could be using overlapped block matrices along the diagonal. However, having more input LD matrices will results in more parameters in deep learning models to train. Therefore, how to keep the balance between reducing potential information loss and how to develop strategies to efficiently handle genome-wide LD matrices as input will be one of our future research directions. Furthermore, to address the limited sample size problem, one potential approach is to use AI-based tools [e.g., TabDDPM (Kotelnikov et al., 2024)] to generate synthetic tabular data. Nevertheless, such approaches require rigorous validation before they can be widely applied.

While Alzheimer’s disease is a polygenic disorder involving numerous loci across the genome, our real data analysis focused on SNPs adjacent to the APOE gene. This region was chosen because of its well-established and strong association with AD, as well as to reduce the computational burden of training deep learning models on genome-wide data. As a result, the analysis serves primarily as a proof-of-concept, demonstrating the model’s ability to capture nonlinear SNP–phenotype relationships within a biologically relevant region. Future work will extend this approach to genome-wide analyses, which will provide a more comprehensive assessment of the model’s predictive ability for polygenic traits. Such extensions will also enable a direct comparison of predictive performance and computational efficiency with polygenic risk scores and other nonlinear models, including kernel-based approaches.

Although the focus of this paper is to compare the performance of deep learning models when only LD matrices are available as inputs with that when individual-level SNP data are available, we would like to briefly discuss the difference between our approach and PRS-based methods, since PRS also relies on genetic summary data. In general, a polygenic risk score for a disease is obtained by aggregating the effects of SNPs across the genome, where the effect sizes of individual SNPs are estimated from genome-wide association studies (GWAS). Therefore, an implicit assumption underlying PRS is that SNP effects are additive and linear, which prevents PRS from capturing nonlinear genetic effects or SNP–SNP interactions (Elgart et al., 2022). In contrast, the proposed deep learning framework learns nonlinear, high-dimensional mappings from SNPs or LD matrices to the phenotype directly, enabling it to capture complex genetic architectures such as SNP–SNP interactions, local LD structure, and potential non-additive effects that PRS cannot model.

Recently, there has been extensive research aimed at opening the black box and making deep learning models more interpretable. According to the survey by Räuker et al. (2023), interpretability techniques can be classified into intrinsic and post hoc approaches. Intrinsic techniques involve training models that are inherently more interpretable or possess natural explanations, whereas post hoc methods aim to interpret a model after it has been trained. From a statistical perspective, one post hoc approach to understand a deep learning model is to use the trained model for statistical inference, such as hypothesis testing or variable selection. Many recent studies have explored such possibilities. For instance, multiple hypothesis testing procedures based on neural networks have been proposed in recent years, with some applied to detecting significant disease-related genes (Horel and Giesecke, 2020; Shen et al., 2021; Shen et al., 2022a; Shen and Wang, 2024; Dai et al., 2024). Although this paper mainly focuses on the predictive performance of deep learning models, the explainability of the proposed methods is also an important topic and will be considered in future research.

## Data Availability

The original contributions presented in the study are included in the article/supplementary material, further inquiries can be directed to the corresponding author.
